# Malformations of the sacculus and the semicircular canals in spider morph pythons

**DOI:** 10.1371/journal.pone.0262788

**Published:** 2022-08-15

**Authors:** J. Matthias Starck, Fabian Schrenk, Sophia Schröder, Michael Pees

**Affiliations:** 1 Department of Biology, Ludwig-Maximilians-University Munich, Munich, Germany; 2 Clinic for Birds and Reptiles, University of Leipzig, Leipzig, Germany; 3 Department Biology, Zoology, University of Cologne, Köln, Germany; 4 Klinik für Heimtiere, Reptilien und Vögel, Stiftung Tierärztliche Hochschule Hannover, Hannover, Germany; Laboratoire de Biologie du Développement de Villefranche-sur-Mer, FRANCE

## Abstract

Spider morph ball pythons are a frequently-bred designer morph with striking alterations of the skin color pattern. We created high-resolution μCT-image series through the otic region of the skulls, used 3D-reconstruction software for rendering anatomical models, and compared the anatomy of the semicircular ducts, sacculus and ampullae of wildtype *Python regius* (ball python) with spider morph snakes. All spider morph snakes showed the wobble condition (i.e., twisting movements of the head, impaired locomotion, difficulty striking or constricting prey items). We describe the inner ear structures in wildtype and spider morph snakes and report a deviant morphology of semicircular canals, ampullae and sacculus in the latter. We also report about associated differences in the desmal skull bones of spider morph snakes, which were characterized by wider semicircular canals, ampullae widened and difficult to discern in μCT, a deformed crus communis, and a small sacculus with a highly deviant X-ray morphology as compared to wildtype individuals. We observed considerable intra- and interindividual variability of these features. This deviant morphology in spider morph snakes could easily be associated with an impairment of sense of equilibrium and the observed neurological wobble condition. Limitations in sample size prevent statistical analyses, but the anatomical evidence is strong enough to support an association between the wobble condition and a malformation of the inner ear structures. A link between artificially selected alterations in pattern and specific color design with neural-crest associated developmental malformations of the statoacoustic organ as known from other vertebrates is discussed.

## Introduction

*Python regius*, ball python, is a constrictor snake that reaches a maximum length of c. 180 cm and is native to West and Central Africa. Because of its moderate size and relative ease of captive husbandry it has become a popular terrarium-housed exotic pet among reptile keepers and hobbyists [1]. The large demand for exotic python pets led to a breeding industry, in which a significant driver of growth is the development of novel color/ pattern strains through artificial breeding selection of gene mutations [2, 3]. Among breeders, spider morph pythons are valued because of their unusual pattern and color appearance. However, frequent neurological diseases (wobble syndrome) have been associated with the inherited color pattern [2].

Wobble syndrome is a collective term for various neurological disorders observed in a variety of animals. In spider morph ball pythons the wobble condition may be expressed to different degrees causing side-to-side or twisting movements of the head, impaired locomotion, and difficulty striking or constricting prey items. A specific cause for the wobble syndrome is not known. Hypotheses have been proposed [4], but none has been tested. Because the wobble syndrome that is associated with spider morph python strains includes dysfunction of the sense of equilibrium, we assumed that it is either associated with central nervous defects, peripheral defects of the vestibular organ (including possible defects of the membranous semicircular ducts with the ampullae, and the maculae of saccule and utricule), or vertebrae malformations. Defects of the vestibular organ and vertebrae malformations should be detectable using μCT-imaging. Our study is descriptive and explorative in a sense that we investigate the morphology of individual spider morph pythons with an explicit anamnesis, and compare it to healthy wildtype animals. Details of the anamnesis, clinical examination and intra vitam diagnostics of the snakes studied here have been reported by [5].

## Materials and methods

We studied *Python regius* (Shaw, 1802) wildtype (N = 5) and spider morphs (N = 4). The spider morphs were presented to the Clinic for Birds and Reptiles at the University of Leipzig by private owners to determine the cause of apparent neurological symptoms. Two of the wildtype snakes were from preserved material at the clinic in Leipzig; they were euthanized for unrelated health reasons. Three wildtype snakes were obtained from preserved material at LMU Munich; protocol notes describe them as healthy when killed for other research purpose. Clinical examination and health status including neurological symptoms in spider pythons have been described in detail in [5]. Animals were euthanized by intramuscular injection of ketamine hydrochloride (100 mg/kg; Ketamine 10%, Selectavet, Germany) combined with medetomidine (0.25 mg/kg; Domitor, Vetoquinol, Ismaning, Germany), then applying T61 (2 ml/kg, Intervet, Unterschließheim, Germany) intracardially.

Because of small sample size, a morphometric and statistical analysis of the inner ear structures is not possible. We therefore restrict the current study to a qualitative comparison of wildtype animals and spider morphs.

The study was conducted in accordance with the faculties ethical committee approval (Faculty of Veterinary Medicine; VMF EK 4/2021).

### μCT-imaging

Before scanning all heads were transferred to 70% and 95% ethanol, and then contrasted in 1% iodine solution in 99.5% ethanol [6, 7] for a minimum of two weeks. Entire heads of snakes P12, P13, P14 and Python 70496 were scanned; the head of *Python* 70496 was bisected after μCT-scanning and then the left side was scanned again. Heads of *Python* control animals 1 and 2, and *Python* 67532, 67533, 67894 were bisected before scanning and both side were scanned separately. Heads were scanned at 3 different institutions, (1) Max Planck Institute for the Science of Human History, Department of Archaeogenetics, Jena; SkyScan 2110; resulting image width * height 1100 * 1100 pixel, 20 μm isotropic voxel size; (2) Zoological State Collection, Munich; Phoenix Nanotom (GE Sensing & Inspection Technologies) CT-scanner; 19 μm isotropic voxel size; and (3) University of Vienna; SkyScan 1174 (Bruker); 23.9μm isotropic voxel size. The operator made every possible effort for straight longitudinal orientation of the heads in the scanner.

### 3D-reconstructions

Series of original images were imported into ImageJ [8] (RRID:SCR_003070) and an image stack was created. The image stack was cropped, optimized for brightness and contrast, and saved in tif-format. The tif-stack was then imported using Drishti Import v2.7 and structures of interest (skull bones, bony labyrinth, membranous labyrinth, neuronal structures) were manually segmented in Drishti [9]. Drishti files were imported in Meshlab_64bit_fp v2021.10 [10] and rendered as 3D-models. Figures were labelled and plates were composed using Adobe® Photoshop CS2 9.0 (RRID:SCR_014199). All μCT-image stacks are accessible at MorphoSource.org, project ID: 000430852 (https://www.morphosource.org/projects/000430852).

## Results

### Wildtype

**Fig 1:**
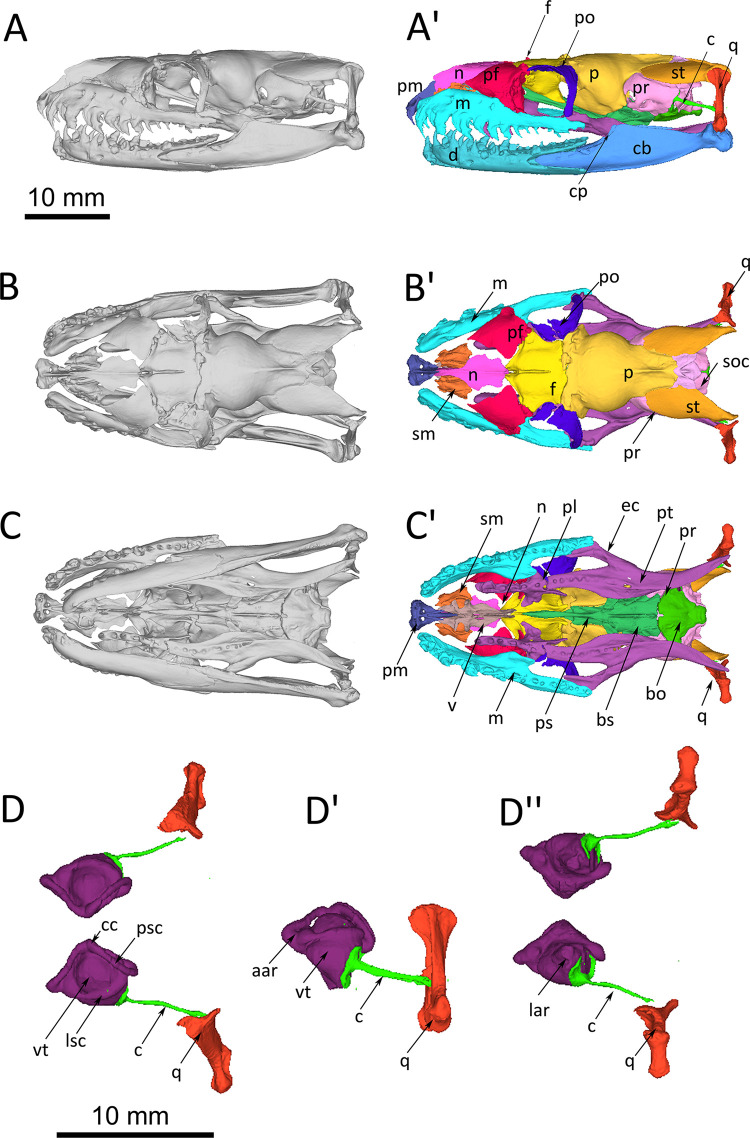
*Python regius* (# P14), orthographic projections of reconstructions of the complete skull of a wildtype individual based on serial μCT-images. For all images left is rostral and right is occipital. The inner ear is housed in the prootic bone and supraoccipital bone. Scale bar in (A) for images A-C’; scale bar in (D) for D-D’. **(A)** Lateral view. (A’) Bones color coded for easy distinction. **(B)** Dorsal view. (B’) With lower jaw removed, bones color coded for easy distinction. **(C)** ventral view. (C’) With lower jaw removed, bones color coded for easy distinction. **(D)** 3D-reconstructed endocasts of the bony inner labyrinth housing the semicircular canals and the sacculus. The columella and the quadrate are elements of the sound transmission apparatus and have been added for orientation. Dorsal (D), lateral (D’) and ventral (D”) view. **Abbreviations:** aar, anterior ampullar recess; bo, basioccipital; bs, basisphenoid; c, columella; cc, crus communis; cb, compound bone; cp, coronoid process; d, dentary; ec, ectopterygoid; f, frontal; lar, lagenar recess; lsc, lateral semicircular canal; m, maxilla; n, nasal; p, parietal; pf, prefrontal; pl, palatine; pm, premaxilla; po, postorbital; pr, prootic; ps, parasphenoid; psc, posterior semicircular canal; pt, pterygoid; q, quadrate; sm, septomaxilla; soc, supraoccipital; st, supratemporal; v, vomer; vt, vestibule.

**Fig 2:**
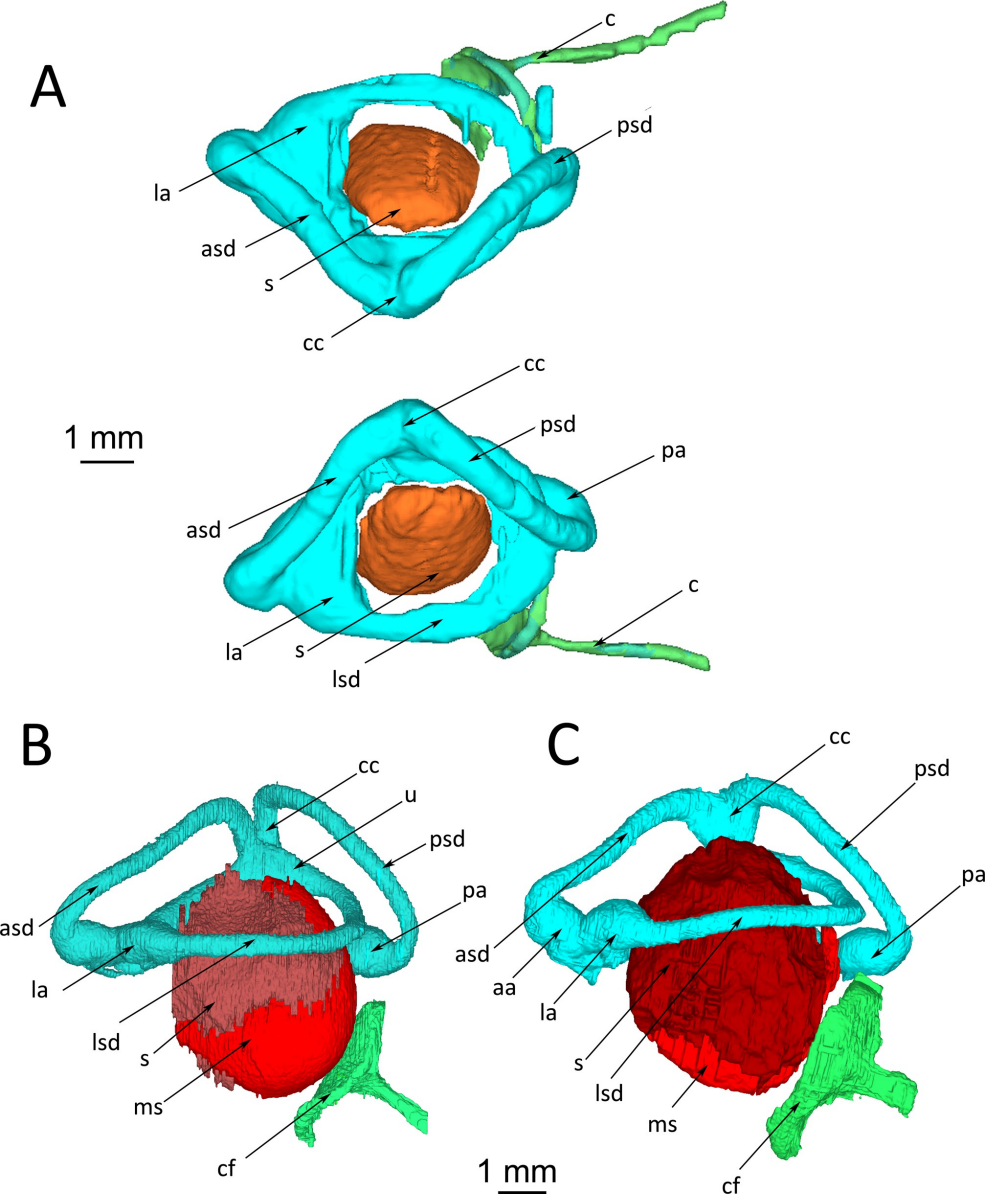
*Python regius*, orthographic projections of 3D-reconstructions of the membranous inner ear of wildtype ball pythons. **(A)** Dorsal view of both inner ears of snake #P14. The reconstruction is based on lower resolution scans of the entire head; therefore, making the reconstruction less sharp than the high-resolution scans in (B) and (C). **(B)** Right ear, control animal #1. **(C)** Right ear, control animal #2. For easy comparisons, images (B) and (C) have been mirrored so that left is rostral and right is occipital. **Abbreviations:** aa, anterior ampulla; asd, anterior semicircular duct; c, columella; cc, crus communis; cf, columella footplate; la, lateral ampulla; lsd, lateral semicircular duct; ms, macula sacculi; pa, posterior ampulla; psd, posterior semicircular duct; s, sacculus; u, utriculus.

The inner ear of healthy wildtype individuals of *Python regius* is housed in the prootic bone and supraoccipital bone (Fig 1A–1C). Prootic and supraoccipital bones are partially overlain by the supratemporal, parietal and otoccipital bones. The columella auris is the single middle ear ossicle. It has a thin ligamentous connection to the quadrate bone. A tympanic membrane and an external ear opening are missing (as in all other snakes). The inner ear consists of the bony labyrinth and the membranous labyrinth. The bony labyrinth forms the semicircular canals (horizontal, anterior and posterior) with their respective ampullar recesses, the vestibulum and the lagenar recess (Fig 1D). The membranous labyrinth resides inside the bony labyrinth and forms the semicircular ducts with their respective ampullae, sacculus, utriculus, lagena and the ductus endolymphaticus (Figs 1 and 2). Sensory epithelia are located in each ampulla, the sacculus, the utriculus and the lagena. Fig 1D documents reconstructed endocasts of the bony labyrinth while Fig 2 documents reconstructions of the membranous semicircular ducts and the sacculus. The sacculus is large and spherical, and reaches dorsad medial to the horizontal semicircular canal. The ampullae are distinct structures at the terminus of each semicircular duct and the utriculus is distinct at the crus communis. The lagena is at the ventro-medial side of the sacculus and not seen in the lateral and dorsal views presented in Fig 2.

**Fig 3:**
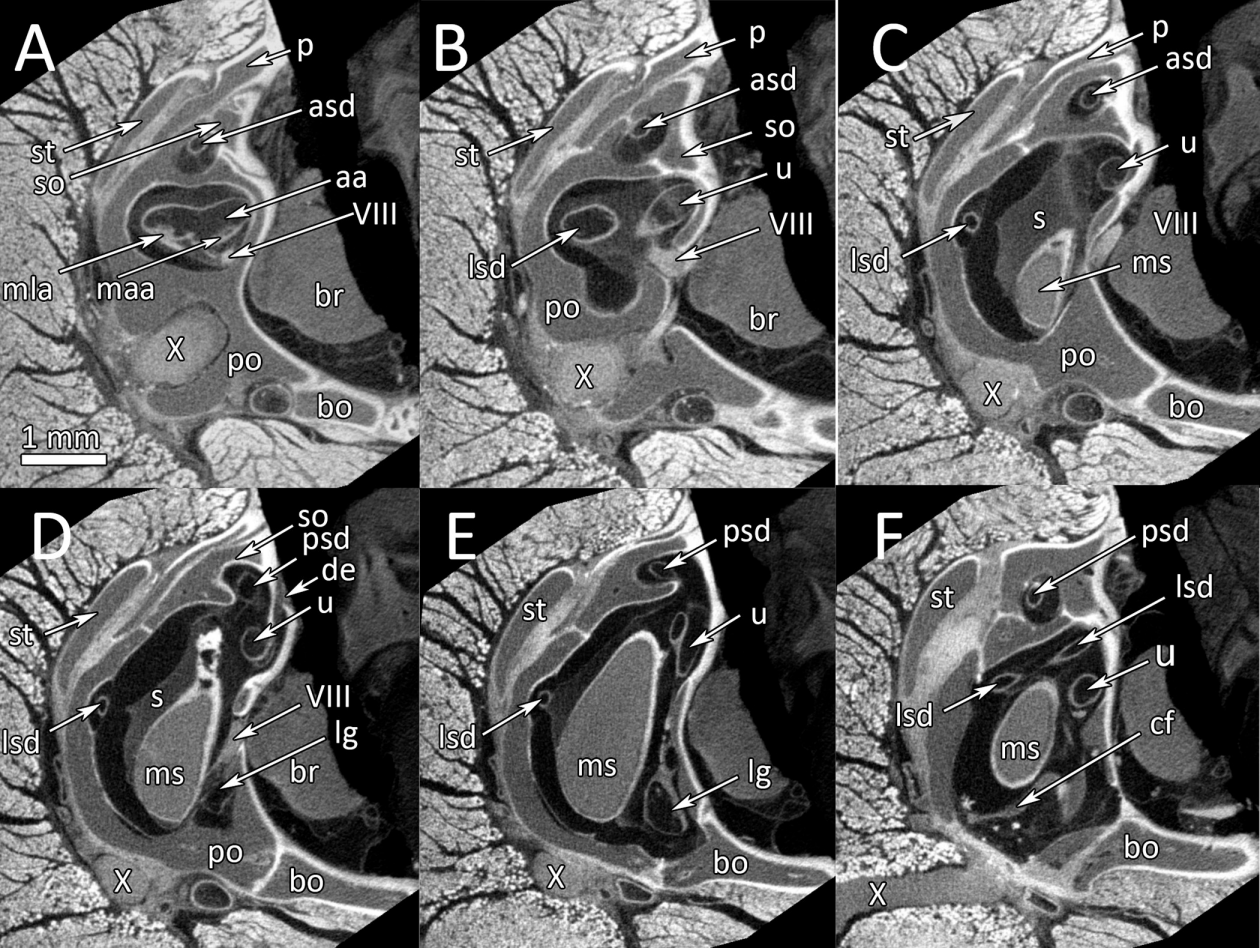
*Python regius*, serial μCT images from rostral to occipital through the inner ear in a wildtype ball python (control animal #1). **(A)** Image #199. **(B)** Image #225. **(C)** Image #283. **(D)** Image #315. **(E)** Image #374. **(F)** Image #461. Scale bar in (A) applies to all images. **Abbreviations:** aa, anterior ampulla; asd, anterior semicircular duct; bo, basioccipital; br, brain; cf, columella foodplate; de, ductus endolymphaticus; lg, lagena; lsd, lateral semicircular duct; maa, macula anterior ampulla; mla, macula lateral ampulla; ms, macula sacculi; p, parietal; po, prootic; psd, posterior semicircular duct; s, sacculus; so, supraoccipital; st, supratemporal; u, utriculus; VIII, nervus stato-acusticus; X, nervus vagus.

μCT-images of the inner ear structures are sufficiently detailed for differentiation of the sensory epithelia and the membranous semicircular canals. Fig 3 documents an anterior-posterior series of selected μCT-images of the right inner ear of a healthy wildtype individual (a 3D-reconstruction of the inner ear of this wildtype snake #1 is shown in Fig 2B). The membranous semicircular ducts are clearly recognizable in all images. They are surrounded by perilymph. In Fig 3A, the anterior ampulla and the lateral ampulla with their respective macula and the branch of the VIIIth nerve are visible. The following images show the lateral (horizontal) and the anterior semicircular canals as well as the utriculus. Fig 3C through F shows μCT-sections through the sacculus. The endolymphatic space and the macula sacculi can easily be differentiated because of their different X-ray contrast. The sacculus reaches dorsad between the lateral semicircular canal and the utriculus (Fig 3D and 3E). The lagena is a comparatively small extension on the medio-ventral side of the sacculus (Fig 3D and 3E). It cannot be seen in the lateral or dorsal views of the 3D-reconstructions (Fig 2) because it is overlain by the sacculus. The μCT-images also provide details about the ductus endolymphaticus (Fig 3D), which, however, has not been reconstructed in Fig 2.

### Spider morph

**Fig 4:**
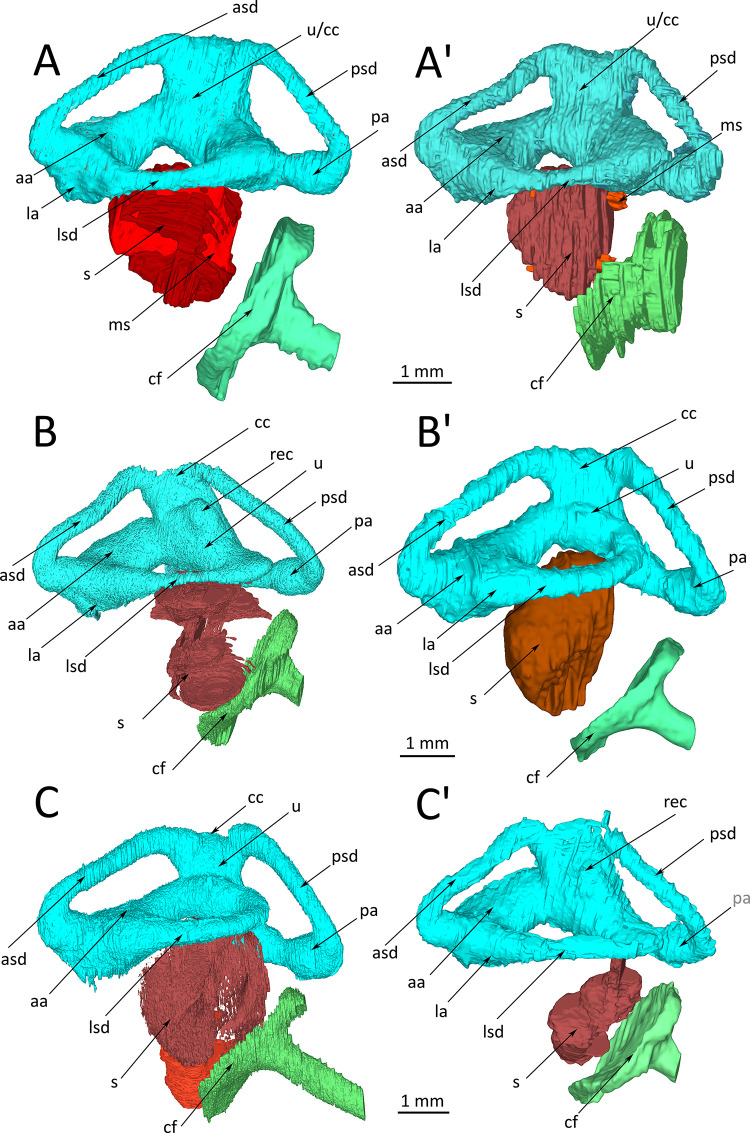
*Python regius*, orthographic projections of 3D-reconstructions of the membranous inner ear of spider morph pythons. For easy comparisons, images (A), (B) and (C) have been mirrored so that left is rostral and right is occipital. **(A)** Right, **(A’)** left inner ear of snake #67532. **(B)** Right, **(B’)** left inner ear of snake #67533. **(C)** Right, **(C’)** left inner ear of snake #67894. **Abbreviations:** aa, anterior ampulla; asd, anterior semicircular duct; cc, crus communis; cf, columella footplate; la, lateral ampulla; lsd, lateral semicircular duct; ms, macula sacculi; pa, posterior ampulla; psd, posterior semicircular duct; rec, recess on the utriculus; s, sacculus; u, utriculus.

The 3D-reconstructions of spider morphs snakes show remarkably different morphology as compared to wildtype animals. The tubes of the semicircular canals are distinctly wider and the ampullae are considerably enlarged as compared to the wildtype animals (compare Figs 2 and 4). The lateral and anterior ampullae appear inflated and, because of their size, merge into each other so that they become difficult to distinguish. An external distinction is not always possible. Also, the utriculus and the crus communis are enlarged. In two individuals (Fig 4B and 4C) the utriculus forms a recess.

The anatomy of the semicircular canals differs within individuals (left-right comparison) as well as between individuals. This is especially evident in the lateral semicircular canal and how it connects to the anterior and posterior semicircular canals (Fig 4); e.g., in snake #67532 the lateral semicircular canal of the left side (Fig 4A’) connects to two enlarged spaces (ampullae) resulting in a largely deformed membranous labyrinth, while on the right side (Fig 4A) it looks more normal, although the ampulla of the anterior canal is also inflated. In snake #67533 (Fig 4B), the lateral semicircular canal of the left side connects directly into the utriculus, while the anterior ampulla is inflated. On the right side of this individual the lateral semicircular canal is thin in the middle part and the utriculus and crus communis are deformed. Individual #67894 (Fig 4C) has a deformed and inflated crus communis and utriculus on the left side, and on the right side the lateral semicircular canal forms a small ring that connects to the anterior semicircular canal.

The most conspicuous difference, which was consistently found in all individuals, concerns the size and morphological integrity of the sacculus. The sacculus is distinctly smaller, never reaches through the opening of the circle formed by the lateral semicircular canal, and is not spherical but deformed. The left and right side of individuals may differ in sacculus structure; this is quite obvious when comparing Fig 4B and 4B’ or Fig 4C and 4C’.

**Fig 5:**
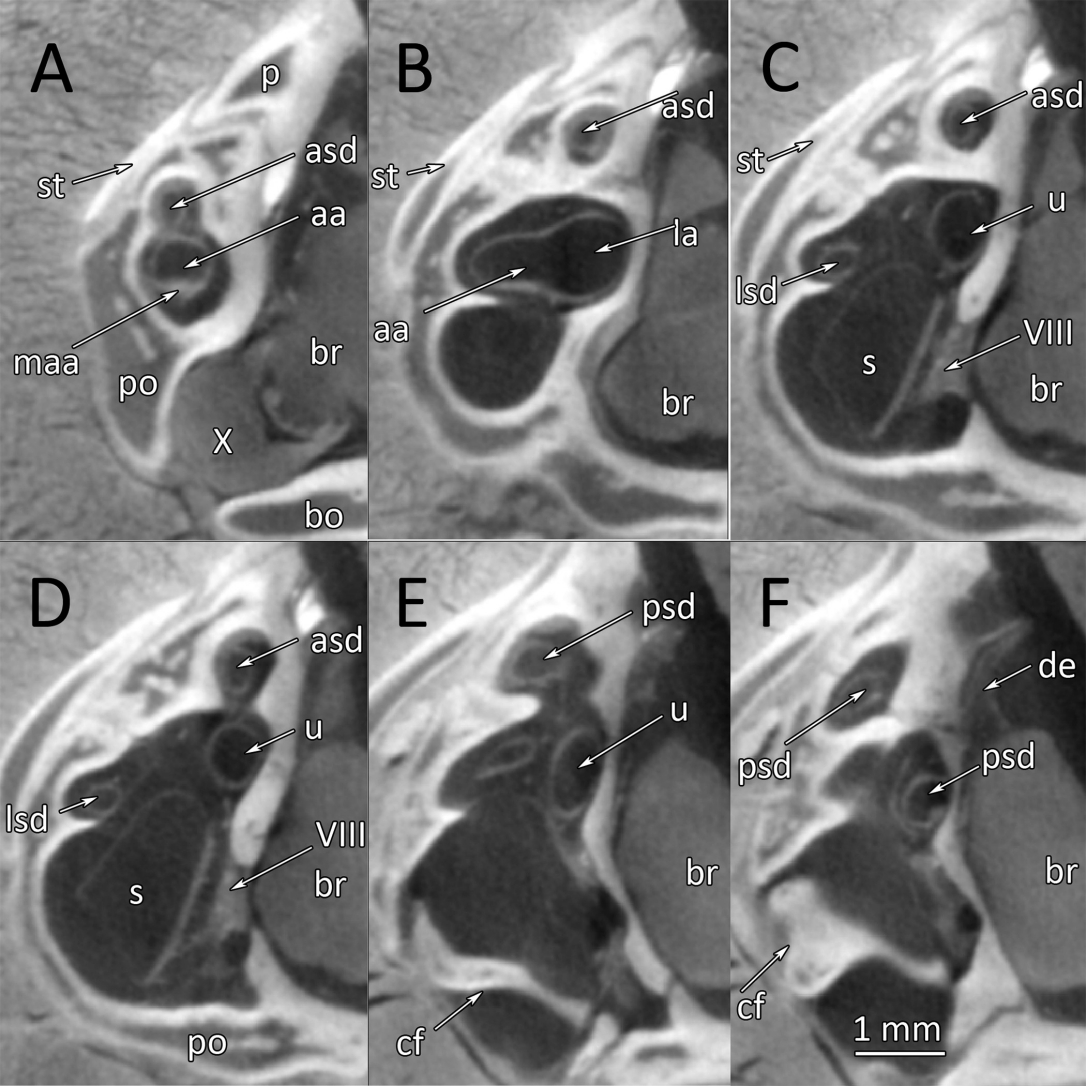
*Python regius*, serial μCT-images from rostral to occipital through the semicircular canals and the sacculus of a spider morph python (snake #67533). **(A)** Image #463. **(B)** Image # 382. **(C)** Image #355. **(D)** Image #345. **(E)** Image #275. **(F)** Image #258. Scale bar in (F) applies to all images. **Abbreviations:** aa, anterior ampulla; asd, anterior semicircular duct; bo, basioccipital; br, brain; cf, columella footplate; de, ductus endolymphaticus; la, lateral ampulla; lsd, lateral semicircular duct; maa, macula anterior ampullae; p, parietal; po, prootic; psd, posterior semicircular duct; s, sacculus; st, supratemporal; u, utriculus; VIII, nervus stato-acusticus; X, nervus vagus.

Exemplar μCT-images document the differences from wildtype animals. Fig 5 represents the same sectional planes as in Fig 3, thus they are directly comparable. Most conspicuous are the inflated appearance of the ampullae, the small size and diffuse morphology of the sacculus, and the lack of a macula sacculi. The serial images in Fig 5 are from the left ear of snake #67533 and may be compared to the reconstructions in Fig 4B’.

**Fig 6:**
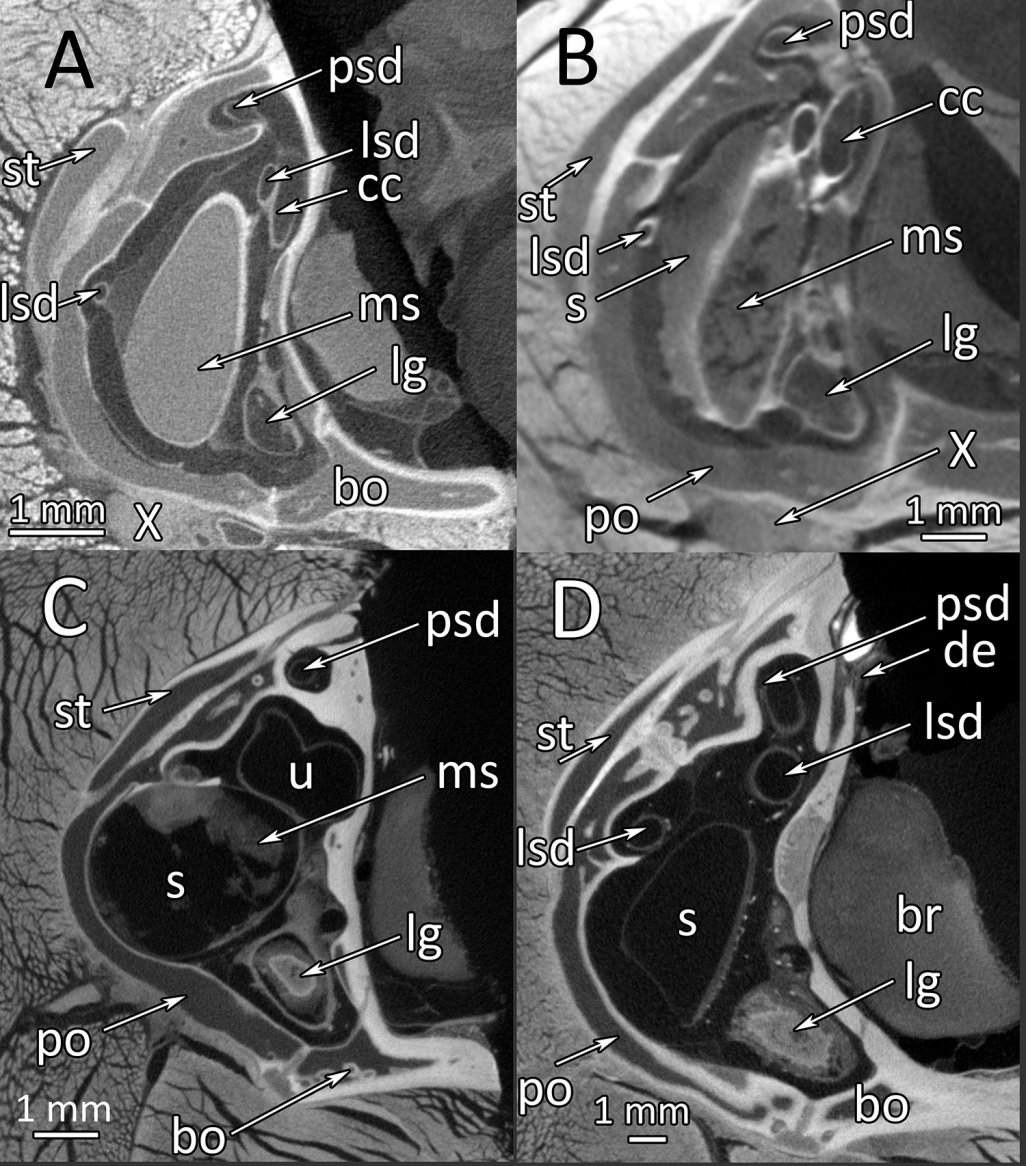
*Python regius*, μCT-images of the sacculus and lagena. **(A)** wildtype snake (control animal #1/image #372). **(B)** Wildtype snake (control animal #2/ image #333). **(C)** Right inner ear of a spider morph snake (#MPI_67533/ image #511). **(D)** Right inner ear of a spider morph snake (# MPI67894 / image #628). **Abbreviations:** bo, basioccipital; br, brain; cc, crus communis; de, ductus endolymphaticus; lg, lagena; lsd, lateral semicircular duct; ms, macula sacculi; po, prootic; psd, posterior semicircular duct; s, sacculus; st, supratemporal; u, utriculus; X, nervus vagus.

Intra- and interindividual differences have been highlighted above and can also be found in μCT images of the other snakes studied. Fig 6 is a direct comparison of a cross-sectional plane through the sacculus and lagena of two wildtype snakes (Fig 6A and 6B) and two spider morph snakes (Fig 6C and 6D). The macula sacculi is distinct and clear in the wildtype animals. The freckling of the macula in Fig 6B is most probably caused by a fixation artifact, however, the originally coherent macula structure can still be recognized. In spider morph snakes, the macula sacculi is either disorganized in a small sacculus (Fig 6C) or completely missing (Fig 6D). In spider morph snakes, the lagena is filled with a structure of low X-ray contrast. It would require histology to determine the nature of this content.

### Desmal skull bones

**Fig 7:**
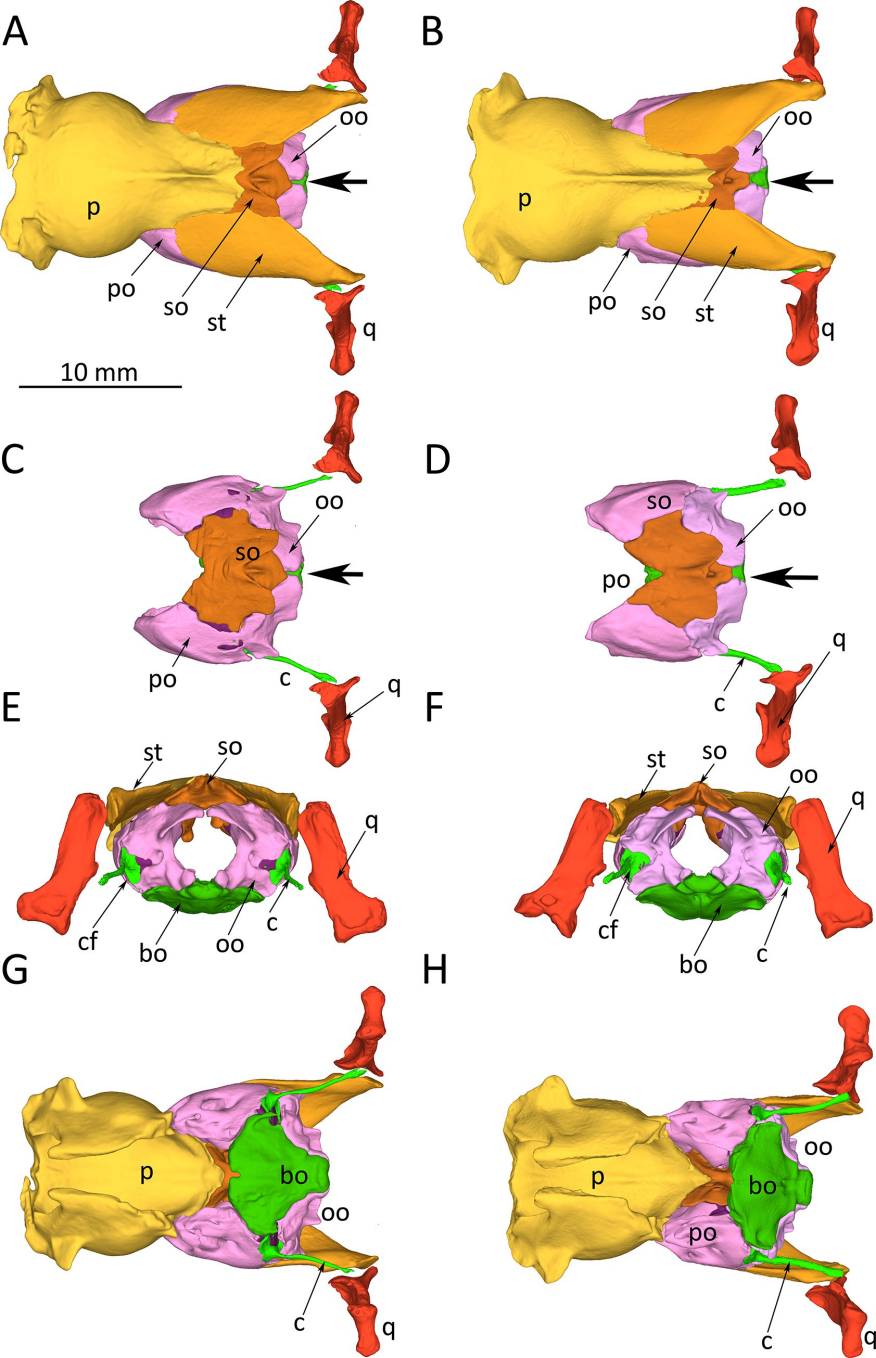
*Python regius*, orthographic projections of reconstructions of the occipital region of skulls of a wildtype individual (left image column, snake P14) and a spider morph python (right image column, snake #70496) based on serial μCT-images. **(A)** Dorsal view of the occipital region of the skull of a wildtype snake. **(B)** Dorsal view of the occipital region of the skull of a spider morph snake. **(C)** Dorsal view of the occipital region of the skull of a wildtype snake, parietal and supratemporal bones removed so that prootic and supraoccipital bones are exposed. **(D)** Same view as (C) of a spider morph snake. **(E)** Occipital view of the skull of a wildtype snake. **(F)** Same view as (E) of a spider morph snake. **(G)** Ventral view of the skull of a wildtype snake. **(H)** Same view as in (G) of a spider morph snake. **Abbreviations**: bo, basioccipital; c, columella; cf, columella footplate; oo, otoccipital bone; p, parietal; po, prootic; ps, parasphenoidone; q, quadrate; so, supraoccipital; st, supratemporal; the large black arrow points to differences in the gap between contralateral otoccipitals. Scale bar in (A) applies to all images.

We next examined the desmal skull bones to determine if there were differences associated with the distinct morphology of the semicircular ducts and sacculus in spider morph snakes. However, due to material limitations, we could compare only one pair of wildtype and spider morph snakes. We found minor differences in the size and morphology of the supraoccipital bone and the otoccipital bones. In the spider morph snake, the otoccipitals gaped distinctly wider than in the wildtype snake (Fig 7A–7F). The crest of the supraoccipital was wider and more robust in wildtype snakes as compared to spider morphs. We could not find any distinct morphological differences on the ventral side of the skull. (Fig 7G and 7H). While these findings in a paired comparison of individual snakes have only the value of a case study, we consider them indicative of true differences between groups (wildtype vs. spider morph) because all other skull bones converge on the same morphology and do not show such remarkable differences.

## Discussion

We compare wildtype and spider morph individuals of *Python regius*. All wildtype individuals were healthy and showed no signs of wobble syndrome, while all spider morph individuals showed noticeable neurological problems that were described as “wobble syndrome” [5]. Sample size was primarily limited by access to material. Breeders are usually reluctant to present their animals to the clinic because they are concerned about health issues that would prevent them from breeding. Over a period of 2 years, only 5 spider morph pythons were presented to the clinic. Sample size in this study is also limited by the time-consuming procedure of 3D-reconstruction, which requires manual segmentation of all structures of interest.

μCT-imaging, although via different scanners and producing different contrast quality, renders cross-sectional images that are sufficiently detailed for a 3D-reconstruction of the membranous semicircular canals, ampullae and sacculus, sensory structures (maculae), as well as the bones housing the inner and middle ear structures. An overview on the ophidian membranous labyrinth exists [11] but not for *Python regius*; however, these data were based on macroscopic dissections and are difficult to compare with the μCT-imaging presented here. All recently published papers using μCT-imaging report on the bony labyrinth [12–15]. A study by [16] focused on the hearing capabilities of healthy *Python regius*. This study presented also a 3D-reconstruction of the bony labyrinth and vestibule (including the macula sacculi), columella, and quadrate. All published μCT-data is fully consistent with our reconstructions. Therefore, we consider this independent support that the methods and documentation used here are fully appropriate to describe and record differences in morphology between wildtype animals and spider morph snakes. Our data comport with classical as well as modern (μCT of the bony labyrinth) documentations of the ophidian inner ear structures.

We find distinct morphological differences when comparing healthy wildtype snakes and spider morph snakes. In spider morphs, the cross-sectional diameter of the semicircular canals appears wider and the ampullae and the crus communis are inflated. The shape of the semicircular canals varies (intra- and interindividual) in the points of fusion and the diameter of the circle formed by each of the canals. Besides the structural differences, the inter- and intraindividual variability appears higher in spider morph snakes than in wildtype snakes.

Most conspicuous is the morphological difference in the sacculus when comparing wildtype and spider morphs. As reported above, the sacculus of spider morph snakes is distinctly smaller, deformed, and lacks a coherent macula. Because the sacculus and the ampullae of the semicircular canals are organs of equilibrium, it is not surprising that these morphological differences are associated with neurological symptoms including head tilt, tremor, corkscrewing, reduced striking accuracy, and reduced righting reflex (reported in detail in [5]). An independent study by [2] showed that all spider morph pythons were affected by the wobble condition, however, to some variable degree.

### Spider morph python and vestibular disorder

Given the small sample size in our study the results rather are like an “extended case study” and certainly cannot be tested statistically. Therefore, conclusions should be drawn carefully. Concerns may be raised that the sample of spider morph snakes presented to the clinic might be biased because only individuals with wobble syndrome were presented. This may be true, but there are several lines of independent evidence that suggest an association of python spider morph strain with the occurrence of vestibular disorders.

Spider morph pythons have been selected for variation in color pattern and melanocyte density. An association between oto-vestibular dysfunction and melanocyte density (different forms of albinism) has been reported for many albinotic animals including man and arguments for a common causative aetiology related to melanocyte function were mentioned in most of the literature [17–20]. This genetic association has long been known and has resulted in appropriate warning against use of albinotic animals as models in biomedical research [21]. Pleiotropic effects of color-associated mutations in mammals and man have been reviewed by [22]. The authors highlight that the sensory organs and nerves are particularly affected by disorders because of the shared origin of melanocytes and neurocytes from the neural crest. Although the inner ear mainly derives from otic placode material and occipital bones housing the inner ear structures derive from axial mesoderm, an intimate interaction between migrating neural crest cells and otic neurogenesis has been described (for mammals). Specifically, neuroblasts were specified from otic vesicle epithelium, but the ganglion develops in close association with neural crest cells, which give rise to glia [23].

Our study is of an exotic pet species that has not been considered in developmental studies. Even normal healthy hearing function has been studied only once in this species (Christensen et al. 2012). The difficulty of obtaining material and the reluctant cooperation of snake breeders prevents us from providing statistical evidence for an association of spider morph breeds and neurological disorder (but see [2] for an evidence-based approach). However, we provide explicit morphological evidence that four individual spider morph snakes have a morphology of the semicircular canals and the sacculus that is deviant from the wildtype. The deformations of the sacculus, the (partial) lack of the macula sacculi, and inflated morphology of the semicircular canals suggest that the sense of equilibrium does not function properly in individuals with such deviant inner ear morphology. The conclusion is straightforward that the observed wobble syndrome in those four snakes is caused by the deviant morphology of their inner ear. The intra- and interindividual variability of the deviant morphology might be related to the described variability in the expression of the wobble condition (from light to severe). Only more exhaustive, non-invasive investigations of the stato-acoustic system in spider morph pythons can provide a sufficiently large sample size to support an association of deviant inner ear morphology of snakes with wobble syndrome.

## Conclusions

We report a deviant morphology of the inner ear in four individual spider morph pythons, which all showed the wobble condition. The deviant morphology probably massively affected the statoacoustic sense. Together with previously published evidence that all spider morph pythons are, to varying degree, affected by the wobble condition, and together with the long-known association of color-mutants with otoacoustic diseases, we suggest that breeding for alterations in pattern and specific color design like spider morph pythons might be linked to neural-crest associated developmental malformations of the statoacoustic organ. The observed intra- and interindividual variation might account for the variability in severity of the wobble condition. Of course, it will require more systematic investigations and a larger sample size to provide evidence of a statistical correlation between clinical symptoms of wobble disease and the extent of vestibular disorder.

## Supporting information

S1 File (DOCX)
