# Supplementary material for: Malformations of the sacculus and the semicircular canals in spider morph pythons
Source: PLoS One. 2022 Aug 15;17(8):e0262788. doi: 10.1371/journal.pone.0262788 (PMC9377635; doi:10.1371/journal.pone.0262788)
Supplement: S1 File — (DOCX) [file pone.0262788.s001.docx]

S1 Original image stacks have been uploaded to MorphoSource (MorphoSource.org, project ID: 000430852). The direct link is: <https://www.morphosource.org/projects/000430852> (the data is also available using authorname "Starck" as search item on MorphoSource). The project contains the following files:

1 µCT-image stack wildtype python control 1

2 µCT-image stack wildtype python control 2

3 µCT-image stack wildtype python 12

4 µCT-image stack wildtype python 13

5 µCT-image stack wildtype python 14 (whole skull)

6 µCT-image stack wildtype python 14 (occipital region of skull)

7 µCT-image stack spider morph python 67532

8 µCT-image stack spider morph python MPI67532

9 µCT-image stack spider morph python 67533

10 µCT-image stack spider morph python MPI67533

11 µCT-image stack spider morph python 67894(left)

12 µCT-image stack spider morph python MPI 67894(right)

13 µCT-image stack spider morph python 70946
